# Autophagy in Diabetes Pathophysiology: Oxidative Damage Screening as Potential for Therapeutic Management by Clinical Laboratory Methods

**DOI:** 10.3389/fcell.2021.651776

**Published:** 2021-04-27

**Authors:** Ezekiel Uba Nwose, Phillip Taderera Bwititi

**Affiliations:** ^1^School of Community Health, Charles Sturt University, Orange, NSW, Australia; ^2^Department of Public and Community Health, Novena University, Kwale, Nigeria; ^3^School of Biomedical Sciences, Charles Sturt University, Wagga Wagga, NSW, Australia

**Keywords:** biomarkers, cause-and-effect, erythrocyte, laboratory tests, oxidative stress

## Introduction—Phenomenology

**Figure 1:**
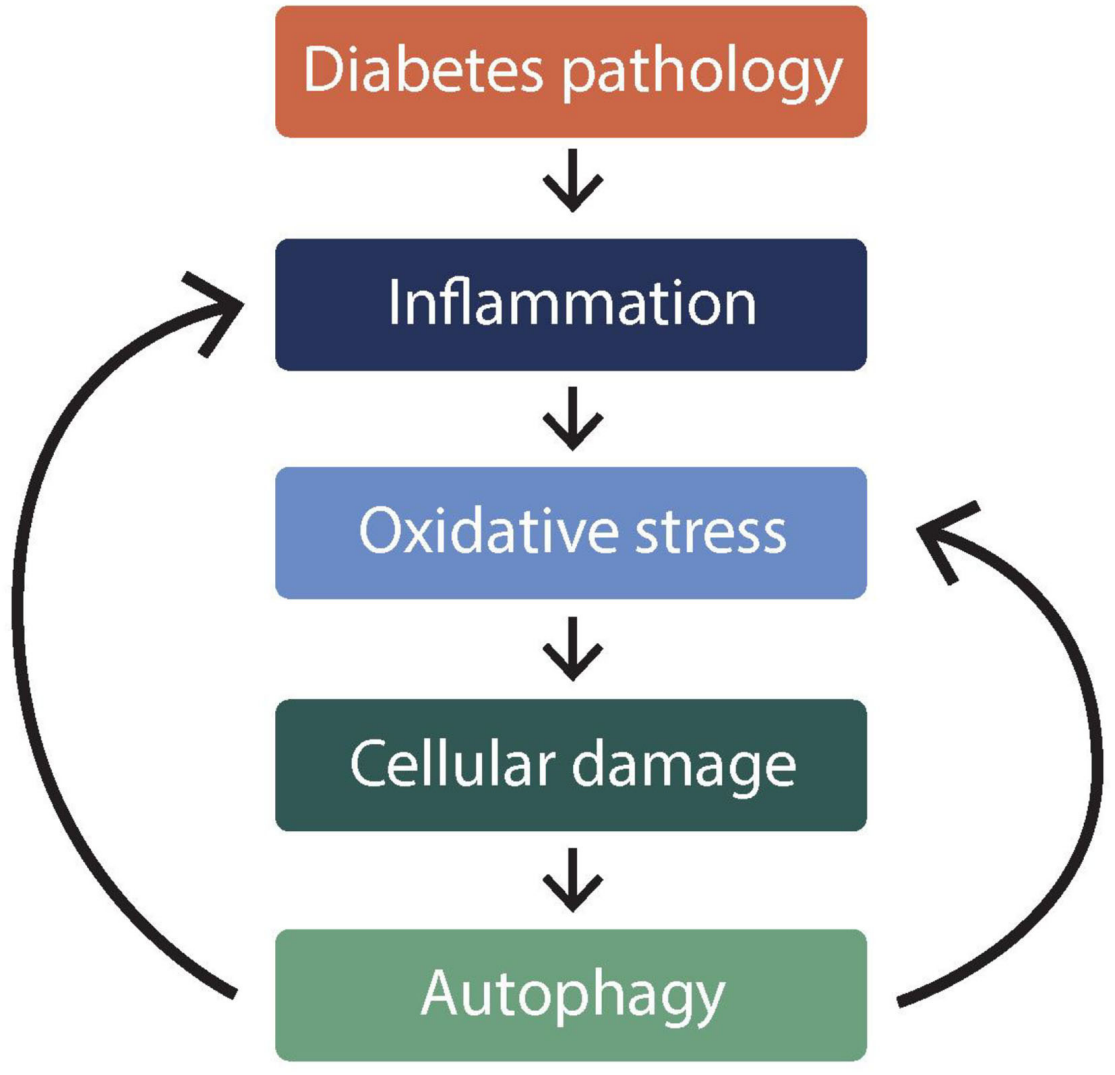
Illustration* of relationship between autophagy and oxidative stress in diabetes mellitus. *This figure illustrates how autophagy is inseparable from inflammation and oxidative stress phenomena. Figure shows that autophagy is caused by (downward arrows), but it also exacerbates (backward arrows), inflammation, and oxidative stress in diabetes.

Autophagy or auto-phagocytosis is self-phagocytosis of tissues and cellular materials i.e., “self-maintenance.” While phagocytosis normally refers to the innate immune process of white blood cells engulfing foreign bodies such as infectious materials, autophagy is a regulatory phenomenon of dysfunctional cellular components being removed (Kobayashi, 2015). Autophagy is inseparable from inflammation and oxidative stress phenomena (Turkmen, 2017); which are intricately involved in pathophysiology of diabetes mellitus (DM) (Muriach et al., 2014). The relationships between phenomena is shown in Figure 1.

Autophagy in association with oxidative stress is involved in pathophysiology. For instance, in the non-modifiable aging process, autophagy is involved in the associated oxidative stress and this can be assessed by glycation end-products as well as indices of lipid oxidation such as malondialdehyde (Moldogazieva et al., 2019). What is yet to be articulated for clinical translation in terms of laboratory assessment of autophagy is the concept of oxidative stress screening. A recent review highlights cell culture and electron microscopy methods (Yoshii and Mizushima, 2017) but not blood tests for oxidative stress indices. Thus, the gap between knowledge and practice are the apparent lack of acknowledgment of oxidative damage interplay between autophagy and metabolic diseases.

The objective of this paper is to bring to the fore the way in which clinical laboratory tests for oxidative stress panel can be used to assess autophagy in metabolic syndrome, especially the relevance of tests from different thematic sub-panels to establish cellular damage in metabolic syndrome. In this objective, cognizance is taken that metabolic syndrome is a constellation of diabetes and its cardiovascular complications including dyslipidaemia factor. For instance, abnormal cholesterol can exacerbate oxidative stress to increase autophagy in diabetes.

This opinion paper is organized in four sections. First three sections cover “causes, consequences, and therapeutic challenges” in terms of oxidative stress, effects on vascular physiology, and implications for management by laboratory methods, respectively. A brief fourth section is on availability of clinical laboratory tests for oxidative stress panel and how to interpret the results in terms of autophagy-inflammation interplay.

## Causes of Autophagy in Diabetes—Erythrocyte Oxidative Stress (EOS) Perspective

Cellular oxidative stress can induce mitochondrial damage, which then requires autophagy to maintain homeostasis (Lee et al., 2012). Oxidative stress is a disturbance of the physiological control of oxidant/antioxidant (redox) balance, in which oxidants become dominant. It is a state in which a cell experiences alteration of cellular components, due to exposure to free radicals and other reactive oxygen species (ROS) beyond its antioxidant capacity (Sies, 1991). Redox reactions are essential for cellular functions such as the utilization of chemical energy from nutrients for the production of adenosine triphosphate. However, excessive oxidants expose cells including erythrocytes to oxidative stress (Kuhn et al., 2017).

EOS is a type of cellular oxidative stress, which arises from over-exposure of the cellular components of red blood cells to various ROS (Richards et al., 1998). It is a situation whereby the erythrocyte's functional mechanisms are impaired or overwhelmed by alteration in the normal metabolic and/or physiological activities that generate ROS (Taniyama and Griendling, 2003). Although the red cell has an efficient antioxidant system for the normal levels of oxidants generated in its membranes, the oxidant challenge can exceed the capacity of the antioxidant system (Fung and Zhang, 1990).

There is a tendency for imperfect reduction of oxygen in the mitochondrial electron transport systems e.g., the leak of superoxide radicals (Maxwell and Lip, 1997). The cascade reaction induced by the superoxide radicals involves antioxidant function of “reduced” glutathione (GSH) that leads to reduction in concentration and exacerbates EOS (McMullin, 1999; Boada et al., 2000; Ulusu et al., 2003). Thus, there are three possible sources of ROS that predispose the erythrocytes to oxidative stress:

- There exist special channels on the membrane by which superoxide radicals permeate the erythrocyte from the mitochondria of other cells (Richards et al., 1998). This is more so during hyperglycaemia or dyslipidaemia (Taniyama and Griendling, 2003). A discussed how EOS is strongly implicated in diabetes and its cardiovascular complications (Nwose et al., 2007a). Laboratory-based investigations have also reported on erythrocyte morphology or oxidative stress being associated with oxidative stress (Parthiban et al., 1995; Nwose et al., 2009; Gyawali et al., 2015). What is being advanced here is the EOS interplay with autophagy in metabolic syndrome (Figure 1).
- Secondly, the erythrocyte can paradoxically become oxidatively stressed from normal physiological processes (Kuhn et al., 2017). Due to the role of erythrocytes in oxygen transport and the presence of redox-active hemoglobin molecules, they generate pro-oxidant radicals by the Fenton reaction.
- Thirdly, there is hyperglycaemia-induced oxidative stress. Besides glycolysis being associated with oxidative stress in diabetic cardiovascular physiology (Zinman, 2003; Brownlee, 2005), there are points of pro-oxidant production when the erythrocyte is utilizing glucose to generate energy in the pentose phosphate pathway (Nwose et al., 2007a).
- Cellular oxidative stress can induce mitochondrial damage, which then requires autophagy to maintain homeostasis.

Therefore, point of emphasize is that fragments of damaged red blood cell materials are removed from the system by the phagocytosis function of the spleen—the basic cleaning function of the blood by spleen. In the context of splenectomy, red blood cells tend to acquire autophagic vacuoles (Holroyde and Gardner, 1970). Current research is yet to translate the basic science that EOS is followed by splenic autophagy of the damaged red blood cells. Therefore, what is being brought to the fore is a measurable perspective of autophagy in terms of EOS that is integral to diabetes and associated metabolic syndrome indices.

## Consequences—Potential Implications of Autophagy-EOS Interplay in Diabetes

There can be autophagy of pancreatic beta-cells (Marasco and Linnemann, 2018), and this has implications, but is not the focus of this discussion. Mitochondrial oxidative stress and autophagy are implicated in diabetes (Muriach et al., 2014); and although red blood cells lack mitochondria, there are potential effects on glucose metabolic pathways. Whether in glycolysis or pentose phosphate pathway, the physiology to meet the cellular need of energy in the erythrocyte is associated with the propensity to deplete GSH content, which in turn leads to EOS. The aberrant state of EOS is of clinical importance in diabetes (Nwose et al., 2007a), especially because hyperglycaemia exacerbates oxidative stress (Yano et al., 2004).

In the diabetes pathophysiology, GSH level is depleted as it is converted to oxidized glutathione. This leads to reduced erythrocyte antioxidant capacity including impaired vitamin E regeneration system (Nwose et al., 2008a), which feedforward to constitutes a possible cause of EOS (Nwose et al., 2007a). Methaemoglobin reductase activity is the other pathway, which impairs GSH functions and potentially complicates the entire vitamin E recycling system (Nwose et al., 2008a).

Thus, diabetes is associated with a decrease of erythrocyte GSH level, which translates to antioxidant imbalance of the cell. Prolonged impairment of the vitamin E recycling in the red blood cell membrane amounts to EOS that damages the cell (Nwose et al., 2008a). What is being brought to the fore is that hyperglycaemia-induced EOS can cause autophagy and may complicate a cause-and-effect phenomenon in diabetes (Figure 1). Therefore, the laboratory perspective is to view autophagy and associated challenges as follows:

- Causes—oxidative stress
- Consequences—effects on cardiovascular physiology
- Therapeutic challenges—implications for management by oxidative stress panel screening.

## Therapeutic Challenges

### Implications for Management by Laboratory Methods

For over two decades, research has demonstrated that changes in erythrocyte antioxidant and haem components in DM lead to complications such as cardiovascular diseases (Dominguez et al., 1998; Dumaswala et al., 2001; Memisogullari et al., 2003), but the question is how EOS is involved in macrovascular complications of DM. As illustrated (Nwose et al., 2007a), EOS may effect macrovascular events including increased blood viscosity, hypercoagulation, and endothelial dysfunction. It is noteworthy that these vascular events constitute Virchow's triad, which has been a subject of research (Makin et al., 2002; Lowe, 2003; Nwose et al., 2014); and shrouded in discussion (Bagot and Arya, 2008; Dickson, 2009; Malone and Agutter, 2009). There is also the effect of tissue hypoxia as discussed that can lead to high blood pressure hence exacerbate metabolic syndrome.

#### Endothelial Dysfunction Exacerbated by Metabolic Syndrome

There is knowledge of hyperglycaemia-induced endothelial dysfunction (De Vriese et al., 2000; Zinman, 2003); and that diabetic dyslipidaemia (component of metabolic syndrome) initiates a chronic inflammatory reaction that results in endothelial damage, which culminates in endothelial dysfunction such as atherosclerosis and coronary artery disease (Gonzalez and Selwyn, 2003). It is established that oxidative stress results in endothelial dysfunction, which has plasma homocysteine levels as clinical index (Dumaswala et al., 2001; Nwose et al., 2007a).

#### Blood Viscosity

This is an intrinsic resistance of blood flow in the vascular system (Lowe et al., 1997). Normally, erythrocyte membrane deformability is a physical property that enables cells to change shape and flow with little or no aggregation/friction. When EOS occurs through lipid peroxidation within the membrane, the cell membrane becomes more rigid and less adaptable (Suda et al., 1980). This makes the blood more viscous, which leads to the development of vascular abnormalities including atherothrombosis and endothelial dysfunction that are associated with coronary artery disease (Solans et al., 2000), as well as tissue hypoxia (El-Sayed et al., 2005). The implication in diabetes and dyslipidaemia has been highlighted (Nwose, 2010, 2013; Richards and Nwose, 2010; Nwose et al., 2014). The point advanced here is the potential, as part of oxidative damage indices for screening splenic autophagy of red blood cellular materials after EOS.

#### Imbalance of Coagulation and Fibrinolysis

There are several theories surrounding hypercoagulation in DM. For instance, hypo-fibrinolysis occurring as thrombomodulin-thrombin complex—which is formed on intact vascular endothelium—may activate thrombin-activatable fibrinolysis inhibitor (Yano, 2003). This suggestion is supported by the observation that hyperglycaemia and insulin enhance the synthesis and secretion of plasminogen activator inhibitor type 1 (Kohler and Grant, 2000). These findings imply that fibrinolysis and therefore the generation of D-dimer are reduced in DM. A seemingly opposing theory is that EOS leads to enhancement of events such as increased production of procoagulant tissue factors at the gene level (Brownlee, 2005), which imply that D-dimer changes in diabetes have not been adequately explained.

It has been reported that some coagulation markers such as D-dimer and fibrinogen are elevated in DM (Sommeijer et al., 2004). Preliminary reports have also shown increased D-dimer levels in DM (Nwose et al., 2007b). Therefore, it is advanced that D-dimer constitutes a potential option for oxidative damage screening (Table 1).

**Table 1:** Oxidative damage panel useable in the laboratory as oxidative stress screening.

**Theme**	**Biomarker**	**Expectation in oxidative stress**	**Sample type^**a**^**
Oxidative stress	Glutathione (GSH)	Reduced antioxidant level	Heparin, citrate, EDTA blood (RBCs or plasma, serum)
	Malondialdehyde (MDA)^b^	Increased oxidant level	EDTA plasma, serum, saliva, urine, cell culture extracts, tissue extracts
	Isoprostane	Increased levels	Citrated or heparin plasma, urine
	Methaemoglobin (metHB)	Increased oxidant level	Citrate or heparin plasma, RBC hemolysate
Oxidative stress “cardiovascular” effect	Blood pressure	Increased	Auscultatory, oscillometry, ultrasound
	D-dimer	Increased hypercoagulability	Heparin, citrate plasma, whole blood
	Homocysteine	Increased endothelial dysfunction	Citrate, EDTA plasma, serum, urine
	Whole blood viscosity	Hyperviscosity/slowed blood flow	EDTA whole blood

a *It is expected that lab protocol will establish separate reference range for every sample type used*.

b *Particularly to assess lipid peroxidation that is exacerbated in dyslipidaemia*.

#### EOS-Induced Haemolytic Anemia Exacerbating High Blood Pressure

There is a likelihood that hyperglycaemia-depleted GSH occurs *via* either reduced regeneration due to deficient pentose phosphate pathway (McMullin, 1999), or enhanced hexosamine pathway flux (Brownlee, 2005). The effect is EOS, which leads to a sequence of membrane rigidity and lysis (Fung and Zhang, 1990). The impacts are both hyperviscosity and anemia, respectively. That is, disruption of the normal rheological properties (El-Sayed et al., 2005), which leads to a sequence of anemia, reduced blood/O_2_ supply, ischaemia and subsequently angina, chronic ischaemic heart disease, myocardial infarction or sudden death (McCance et al., 2002). It is noteworthy that the homeostatic response to reduced blood/O_2_ supply involves increase in cardiac output, which may lead to high blood pressure. Hence, evaluation of fluctuations in blood pressure can support oxidative stress panel.

Indeed, anemia is associated with an increased risk of diabetic macrovascular disease or metabolic syndrome *per se* (Thomas et al., 2006). Autophagy is implicated in anemia (Grosso et al., 2017), but what has not been given adequate attention is that:

- EOS compromises the free radical basis of anemia (Dumaswala et al., 2001)
- Anemia is an effect of autophagy-related EOS
- To date, there are no defined signs and symptoms of autophagy for clinical decision making.

### Oxidative Stress Panel Indices for Autophagy—Screening Suggestion

There are limited laboratory methods for autophagy evaluation (Klionsky et al., 2016), especially in diabetes research and practice. Thus, the justification of this paper is to advance the role of oxidative stress panel indices for the screening of autophagy in people living with diabetes. For instance, this could be used to monitor therapeutic management of cardiovascular complications of diabetes.

Diagnostic laboratory markers include the traditional risk evaluation markers such as cholesterol and glucose profiles. Emerging biomarkers include C-reactive protein, D-dimer, and homocysteine (Tracy, 2003; Ridker et al., 2004), as well as oxidative stress indices that include GSH and MDA (Tsimikas, 2006; Gyawali et al., 2015; Nwose et al., 2018; Lubrano et al., 2019). Uric acid and albumin levels have been considered (Bwititi et al., 2012). Pending clear definition of the signs and symptoms of autophagy, it is recommendable that the common laboratory principle applies whereby more than three positive results from this suite of biomarkers may indicate high levels of autophagy, be adopted.

It is becoming more clear that pharmacological agents to promote autophagy work by mediating oxidative stress (Wu et al., 2020). This implies that autophagy status is associated with degree of oxidative stress. The contribution being made in this paper is that methods for clinical evaluation of oxidative stress used in research can be integrated into clinical practice.

Further, it has been established that oxidative damage must involve reduction in antioxidant status, increase in oxidant levels and evidence of cardiovascular effects such as hypercoagulation, endothelial dysfunction, and blood viscosity that could easily lead to diseases (Nwose et al., 2008b). Thus, laboratory screening of oxidative damage includes indices of oxidative stress indices and their effects (Table 1). Oxidative stress markers listed here are just examples of what is readily available. There are a whole lot of others (Keaney et al., 2003; Frijhoff et al., 2015).

Perhaps, it is pertinent to emphasize that although assessment of EOS and its role in autophagy would be a potential therapeutic screening method, what needs to be addressed is “*if it will be an effective indicator of overall metabolic dysregulation*.” For this reason, it is pertinent to point out the concept of high cholesterol (indicated by lipid profile) being a factor that can exacerbate lipid peroxidation (i.e., oxidative stress) to increase autophagy in diabetes. Also, oxidative damage panel screening will show cardiovascular effect such as increased blood viscosity (Nwose, 2013). Thus, beyond e.g., blood glucose and cholesterol dysregulation, there are effects or subclinical pathophysiological indices.

This perspective recommendation advances the knowledge that oxidative stress induces autophagy (Hariharan et al., 2011; Turkmen, 2017; Gao, 2019; Moldogazieva et al., 2019). This therefore justifies carrying out laboratory tests for oxidative stress. The theme of this special issue, therapeutic challenges in the management of autophagy implies the need for monitoring of treatment using tests that assess therapeutic efficacy. It has recently been highlighted that targeting autophagy is a means to counteract oxidative stress in obesity (Pietrocola and Bravo-San Pedro, 2021). Another advantage is therefore identification of need for change in treatment regimen. For instance, there is indication of possible simultaneous induction of oxidative stress and autophagy in diabetes as well as obese patients (Klionsky et al., 2016). What this opinion paper contributes is the need to look at a panel of tests and the availability of the test methods.

## Conclusion

Current considerations of autophagy acknowledge oxidative stress in the cause-and-effect physiology as well as therapeutic modes of action. However, monitoring by laboratory methods is yet to integrate the available and validated oxidative stress parameters. What this opinion paper brings to fore is that tests for oxidative stress panel can be used to assess autophagy in metabolic syndrome, including diabetes and its cardiovascular complications, at no major cost.

## Author Contributions

EN and PB conceptualized the opinion. EN did the initial draft of main text. PB did the revision as well as the abstract and conclusion. Both authors contributed to the article and approved the submitted version.

## Conflict of Interest

The authors declare that the research was conducted in the absence of any commercial or financial relationships that could be construed as a potential conflict of interest.

## Abbreviations

DM: diabetes mellitus
EOS: erythrocyte oxidative stress
GSH: reduced glutathione
MDA: Malondialdehyde
ROS: reactive oxygen species.
